# The contribution of family physicians to chronic disease management through continuity of care in Ghana

**DOI:** 10.4102/phcfm.v13i1.3220

**Published:** 2021-12-10

**Authors:** Stephen T. Engmann

**Affiliations:** 1 Department of Family Medicine, Manna Mission Hospital, Accra, Ghana; 2 Department of Family Medicine/Polyclinic, Korle Bu Teaching Hospital, Accra, Ghana

**Keywords:** chronic care, chronic disease, family physician, care continuity, management, Ghana

## Abstract

Chronic non-communicable diseases contribute significantly to Ghana’s disease burden. Ghana’s ability to achieve universal health coverage is threatened by the rising burden of chronic non-communicable diseases. There is a high unmet need for cardiovascular diseases care, with primary health care for cardiovascular diseases not being readily available, equitable, or sensitive to the requirements of target populations. The contribution of family physicians in the management of the chronic disease burden through care continuity cannot be overemphasised. This is a short report of the implementation of a chronic care clinic by a family physician in Manna Mission Hospital, which is located in the Greater Accra region of Ghana. Before the implementation, there was no such clinic in the hospital and patients with chronic conditions who visited the facility were sometimes lost to follow-up. The clinic which commenced in January 2019 has provided care for patients with chronic non-communicable diseases to date. The most common chronic diseases managed at the clinic include hypertension and heart failure, diabetes, stroke, asthma, sickle cell disease, and joint disorders. This report gives an account of the contribution of family physicians to chronic disease burden management through continuity of care in a low-resource setting like Ghana.

## Introduction

Chronic non-communicable diseases (NCDs) contribute significantly to Ghana’s disease burden.^1,2^ Ghana’s ability to achieve universal health coverage is threatened by the rising burden of chronic NCDs. Cardiovascular diseases, malignancies, diabetes, chronic respiratory disorders, and sickle cell disease are the most common chronic NCDs in Ghana.^3^ Because of aging, rising urbanisation, and unhealthy lifestyles, the burden of chronic NCDs in Ghana is expected to rise.^3^

There is a high unmet need for cardiovascular disease management in Ghana, with primary health care for such diseases not being readily available, equitable, or sensitive to the needs of the population.^1^ Within the African context, family physicians play a critical role in strengthening primary health care and district health services.^4^ Through clinical leadership and direct patient interaction, family physicians have had a positive impact on chronic disease management.^5^ Family Medicine promotes comprehensive, coordinated, and continuous care to patients and their families.

This short report describes the implementation of a chronic care clinic by a family physician in Manna Mission Hospital, which is located in the Greater Accra region of Ghana. It explains the contribution of family physicians to chronic NCD management, particularly the continuity of care in this low-resource setting.

## Description of context

The Manna Mission Hospital is a health facility situated in Teshie, within the Ledzokuku Municipality of the Greater Accra Region of Ghana. It is a Christian Health Association of Ghana (CHAG) – member organisation and forms part of the municipal health services. Christian Health Association of Ghana is an agency under the Ministry of Health of Ghana and a Network organisation of health facilities owned by Christian Church Denominations. The Hospital serves all communities within its catchment area, and healthcare can be accessed through the national health insurance scheme and other health insurance schemes. The Ledzokuku municipality’s health services face institutional issues such as attrition of health workers, insufficient capacity and capability of various health facilities, and insufficient health infrastructure.^6^ Manna Mission Hospital is a 40-bedded medical hospital that provides both primary and secondary levels of care. Services provided include family medicine which forms part of the outpatient department, general surgery, obstetrics and gynaecology, and paediatric care. The facility is departmentalised with specialists in these fields of medicine. The clinical staff and personnel are usually posted to the facility by the Ministry of Health through the CHAG agency. The facility receives some referrals from neighbouring primary care facilities in the community; however, the municipal hospital is the major referral centre of the municipality. The top 10 outpatient morbidities at the hospital include malaria, upper respiratory tract infections, urinary tract infections, musculoskeletal disorders, hypertension, gynaecological conditions, pregnancy and related complications, skin diseases, diarrhoea diseases, and anaemia (Table 1).

**TABLE 1:** Top 10 outpatient morbidity.

Diagnosis	Number of cases
**2019**	
Malaria	1612
Upper respiratory tract infections	1547
Acute urinary tract infections	925
Musculoskeletal and Joint Pains	769
Gynaecological conditions	352
Hypertension	234
Skin diseases	230
Diarrhoea diseases	220
Acute eye infections	198
Ottitis media	163
All others	3504
**2020**	
Malaria	1647
Acute urinary tract infections	1344
Upper respiratory tract infections	1249
Musculoskeletal and joint pains	495
Gynaecological conditions	396
Pregnancy and related complications	288
Hypertension	220
Skin diseases	218
Diarrhoea diseases	215
Anaemia	166
All others	3699

The implementation of the chronic care clinic was the initiative of the author. This was after a family practice needs assessment in the facility and also the growing need for specialised care for patients with chronic disease. The findings from the assessment of the need for chronic care revealed that there was a poor follow-up, provision of care was sometimes fragmented, and patients were not well-informed about their condition. The rationale for the clinic was to provide specialist care and continuity of care for patients with chronic diseases and also reduce the loss to follow-up in the general outpatient clinic, through the provision of ongoing secondary care for NCDs. This report focuses on continuity of care because it is one of the core principles in family medicine. Furthermore, continuity of care ensures better healthcare outcomes, higher patient satisfaction, and more cost-effective healthcare.^7^

## Chronic care clinic

The chronic care clinic was commenced twice a week in January 2019. Continuous care for patients with chronic NCDs has been provided at the clinic to date. The most common diseases managed at the clinic include hypertension and heart failure, diabetes, stroke, asthma, sickle cell disease, and joint disorders. One day a week is for diabetes and the other day for all other conditions. The majority of the patients have hypertension and diabetes.

The goal of the clinic is to improve the overall health and quality of life of patients. Patients are given appointment dates by nurses who can also reschedule if necessary. The clinic was implemented according to the principles of the chronic care model.^8^ The core elements of this model which were applied at the practice level include service delivery design, self-management and decision support, and clinical information systems. The service delivery design employed was the provision of care by a multidisciplinary team of health professionals for the management of patients both within the facility and in other higher-level facilities when necessary.

Patient educational sessions and motivational counselling were amongst the other interventions used to support self-efficacy and lifestyle modification. Before the coronavirus disease 2019 (COVID-19) pandemic, group health education on chronic diseases was given to all patients at the clinic whilst waiting to be seen. But with the outbreak of the COVID-19 pandemic, the group health education was suspended as a measure to ensure social distancing. Currently, health education and promotion are done during consultations to support self-management.

Following the acquisition of an electronic health record system in September 2020, patient data has become readily available for the clinical team to analyse and support informational continuity during ongoing care. The care team is comprised of the author (a family physician) as the leader of the team, with the support of other medical officers, registered general nurses, a dietician, a pharmacist, laboratory staff, and a clinical psychologist.

## Care continuity and effect on primary health care

Continuity of care is ‘the extent to which a series of discrete healthcare events is experienced by people as coherent and interconnected over time and consistent with their health needs and preferences’.^9^ Increased continuity of care by clinicians is linked to lower death rates, hence the need to prioritise continuity of care.^10^ A personal patient-doctor relationship and continuity of care are major facilitators to the management of patients with multimorbidity.^11^

The management of patients with chronic diseases in this report revolved around an enhanced patient-doctor relationship. Enough time was given for consultations to allow patients to express themselves which helped to address their problems and concerns. This created the opportunity for patient-centred care during clinic consultations. Such relational continuity was intended to build familiarity, trust and respect over time. As doctor-patient communication improved, it became easier for patients to discuss sensitive issues. Over the two years of implementing the chronic care clinic, continuity of care was also maintained through an effective appointment system.

## Reflection on the contribution of family physicians

Through the chronic care clinic, there was better longitudinal continuity through an effective appointment system, better informational continuity through the electronic health record, and better relational continuity through an enhanced doctor-patient relationship where patients get to see the same doctor or care team. Family physicians are trained to apply these principles in their practice.

From my experience, the contribution of family physicians in managing patients with chronic illness even in resource-constrained settings will improve the quality of patient care as well as the efficiency and effectiveness of care. The family physician’s use of a biopsychosocial approach to care can impact behavioural changes that patients need to make. Patient educational sessions have also contributed to making patients more informed about their condition. From the standpoint of healthcare, there is evidence that continuity of care increases the survival of older people and lowers direct overall medical costs.^12^

Within the broader health system, consistent management of these chronic illnesses will improve the overall health and quality of life of patients. Through regular assessments by family physicians, the particular risk to the patient can be determined, and those who are developing complications can be identified for early interventions.

## Conclusion

Chronic NCDs remain a significant disease burden in Ghana. Family physicians by virtue of their training contribute to chronic disease management through improving continuity of care. One way to achieve care continuity can be the creation of chronic care clinics that cater more specifically for patients with NCDs, with improved appointment systems, electronic medical records, and ongoing relationships with care providers.
